# Population Heterogeneity of Diabetes in Indigenous Peoples of the Americas: A Systematic Scoping Review of the Existing Literature

**DOI:** 10.3390/jpm16020116

**Published:** 2026-02-14

**Authors:** Alberto Barcelo, Roy Wong-McClure, Felicia Cañete, Ethel Santacruz, Noelia Cañete, Arise Garcia de Siqueira Galil

**Affiliations:** 1 Department of Public Health Science, Miller School of Medicine, University of Miami, Miami, FL 33141, USA; 2 Departamento de Cínica Médica, Faculdade de Medicina, Universidade Federal de Juiz de Fora, Minas Gerais 36036-330, Brazil; galilarise@gmail.com; 3 Caja de Seguro Social, San José 10104, Costa Rica; 4 Cátedra de Salud Pública y Administración Hospitalaria, Facultad de Ciencias Médicas, Universidad Nacional de Asunción, Asuncion 11001-3291, Paraguay; feliciacanete@gmail.com; 5 Dirección de Vigilancia de Enfermedades No Transmisibles, Dirección General de Vigilancia de la Salud, Ministerio de Salud y Bienestar Social, Asunción 001009, Paraguay; ethel.santacruz@mspbs.gov.py (E.S.);

**Keywords:** indigenous, Amerindians, native Americans, diabetes prevalence, health disparities, population heterogeneity, personalized medicine, precision public health, ethnic diversity, Americas, scoping review, epidemiology

## Abstract

**Background:** In the Americas, the number of people living with diabetes is expected to rise from 92 million in 2024 to 120 million by 2050. Indigenous populations may experience distinct biological, environmental, and sociocultural risk factors; however, they are often treated as a homogeneous group in epidemiological research, and consolidated evidence on diabetes prevalence across diverse Indigenous populations remains limited. This scoping review examines the prevalence of diabetes among Indigenous populations in the Americas. **Methods:** Following PRISMA-ScR guidelines, we conducted a systematic scoping review of population-based studies reporting the prevalence of diabetes among Indigenous adult populations in the Americas. Searches were performed in PubMed and Scopus. Collected data included study location, Indigenous group, population characteristics, diagnostic criteria, and test used and reported prevalence estimates. **Results:** Sixty documents encompassing 73 studies met the inclusion criteria, representing 45,503 individuals from 16 countries between 1975 and 2025. The total number of ethnic groups represented was 111, and 12 studies did not identify a specific ethnic group. Fasting blood glucose (FBG) was the most frequently used diagnostic method, followed by the oral glucose tolerance test (OGTT). Estimates of the prevalence of diabetes varied widely across populations, regions, and time periods. Five studies—from Brazil, Chile, Colombia, Mexico, and Paraguay—did not identify any cases of diabetes. Among studies reporting cases, prevalence ranged from 1 to 70% in North America, 5 to 14% in Central America, and 1 to 29% in South America. **Conclusions:** The prevalence of diabetes among Indigenous populations varied widely across the region, with substantially higher estimates reported in North America than in Central and South America. The decline in published studies in recent years suggests reduced research attention to this topic. The marked heterogeneity identified in this review underscores the need for standardized measurement approaches to support population-specific strategies aligned with personalized care and precision public health.

## 1. Introduction

The global prevalence of diabetes was estimated at 589 million people between the ages of 20 and 79 years, in 2024; this number is projected to increase by 46% over the next 25 years. In the Americas, approximately 92 million people were living with diabetes in 2024, with forecasts predicting an increase to 120 million by 2050 [1].

The rising global burden of diabetes is largely driven by the growing prevalence of type 2 diabetes. This pattern is associated with both modifiable risk factors—such as overweight or obesity, physical inactivity, and poor nutrition—and non-modifiable factors, primarily age and genetic predisposition [1].

Before European contact, Indigenous populations in the Americas likely exceeded 100 million and encompassed thousands of distinct peoples and more than 1000 languages, reflecting exceptional demographic and cultural diversity [2,3,4]. Colonization in the sixteenth century caused a severe demographic collapse, driven by introduced diseases and social disruption, leading to the loss of numerous communities and languages [5,6]. Since the mid-twentieth century, Indigenous populations have gradually recovered, reaching an estimated 100 million people today, although marked heterogeneity persists across regions [7,8,9,10].

Historically, an increased prevalence of diabetes has been documented among Native populations in North America. One of the highest recorded rates has been observed in the Pima Indians of Arizona [2]. For years, research has emphasized the critical contribution of genetic susceptibility to type 2 diabetes, especially when compounded by adverse environmental conditions.

Understanding the scope, heterogeneity, trends, and risk factors of diabetes in Indigenous populations of the Americas is crucial for identifying research gaps and guiding future studies. By summarizing existing data, this systematic review aims to provide an evidence-based foundation to improve health outcomes and reduce disparities. The objective of this study is to identify reports on the prevalence of diabetes among adult Indigenous populations across the Americas.

## 2. Materials and Methods

This systematic review was designed to address the following research question: What is the prevalence of diagnosed and undiagnosed diabetes among the adult Indigenous populations of the Americas?

The search strategy adheres to PRISMA guidelines for systematic reviews (PRISMA checklist in Supplementary Table S8). Specifically, this study follows the Preferred Reporting Items for Systematic Reviews and Meta-Analyses extension for Scoping Reviews (PRISMA-ScR). In accordance with these guidelines, the study protocol is registered with the Open Science Framework (OSF): https://doi.org/10.17605/OSF.IO/CY6NH (accessed on 19 January 2026).

### 2.1. Search Strategy

The literature search was conducted using PubMed and Scopus with the following keywords:

(TITLE-ABS-KEY (indigenous) OR TITLE-ABS-KEY (aboriginal) OR TITLE-ABS-KEY (indian) AND TITLE-ABS-KEY (diabetes prevalence) AND TITLE-ABS-KEY (america) OR TITLE-ABS-KEY (mexico) OR TITLE-ABS-KEY (belize) OR TITLE-ABS-KEY (guatemala) OR TITLE-ABS-KEY (el AND salvador) OR TITLE-ABS-KEY (honduras) OR TITLE-ABS-KEY (nicaragua) OR TITLE-ABS-KEY (costa AND rica) OR TITLE-ABS-KEY (panama) OR TITLE-ABS-KEY (colombia) OR TITLE-ABS-KEY (venezuela) OR TITLE-ABS-KEY (peru) OR TITLE-ABS-KEY (bolivia) OR TITLE-ABS-KEY (ecuador) OR TITLE-ABS-KEY (chile) OR TITLE-ABS-KEY (paraguay) OR TITLE-ABS-KEY (argentina) OR TITLE-ABS-KEY (uruguay) OR TITLE-ABS-KEY (brazil) OR TITLE-ABS-KEY (guyana) OR TITLE-ABS-KEY (suriname) OR TITLE-ABS-KEY (united states) OR TITLE-ABS-KEY (canada) OR TITLE-ABS-KEY (puerto rico) OR TITLE-ABS-KEY (cuba) OR TITLE-ABS-KEY (dominican) OR TITLE-ABS-KEY (dominicana) OR TITLE-ABS-KEY (haiti) OR TITLE-ABS-KEY (jamaica) OR TITLE-ABS-KEY (trinidad) OR TITLE-ABS-KEY (grenada)).

In addition, we conducted an internet search for relevant gray literature, including documents from governmental and academic institutions. We also reviewed citations from the retrieved documents to identify additional reports meeting the inclusion criteria that may have been overlooked in the systematic review. Articles that met the inclusion criteria and were identified in a previous search [11] were also included.

### 2.2. Article Selection

**Figure 1:**
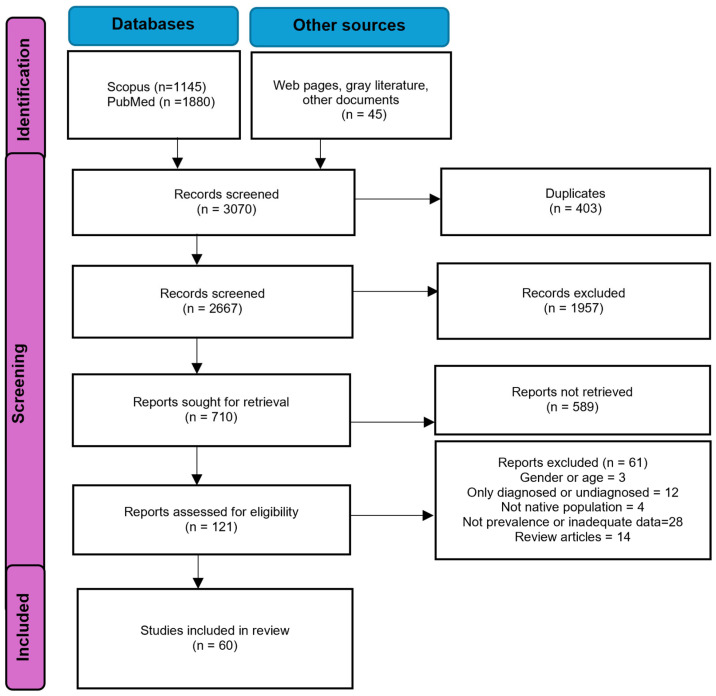
Source: Modified from Page MJ, et al. BMJ 2021;372:n71. https://doi.org/10.1136/bmj.n71 [17]. This work is licensed under CC BY 4.0. To view a copy of this license, visit https://creativecommons.org/licenses/by/4.0/ (accessed on 20 January 2025).

A comprehensive search was conducted for relevant documents from all countries in the Americas, restricted to publications from 1975 to 2025. The search was completed in July 2025. The inclusion criteria required original documents that:

(a) described population-based surveys of the prevalence of diabetes among adult Native or Indigenous subjects of both genders (or a subgroup);
(b) employed an unbiased, adequate population-based sampling procedure (probabilistic sample, cluster sample, or census);
(c) reported prevalence rates in percentages;
(d) included total diabetes prevalence (diagnosed plus newly identified cases) using international diagnostic blood glucose standards and tests available at the time of the study (as shown in Figure 1); and
(e) were published in English, Spanish, French, or Portuguese between 1975 and 2025.

Studies were excluded if they exhibited any source of selection bias, such as non-random samples or clinical series. All retrieved articles were independently reviewed by three reviewers (ES, FC, RWM, NCB, or AB).

### 2.3. Data Extraction

Data were independently extracted by two reviewers, selected from ES, FC, RWM, and NCB. Discrepancies were resolved by a third reviewer (AB) before finalizing the dataset. Extracted data points included country, author, study site, year of study, year of publication, ethnic group, age, sample size, diagnostic test, diagnostic method and values, and prevalence rate.

### 2.4. Data Analysis

The extracted data on the prevalence of diabetes among Indigenous populations in the Americas were analyzed descriptively. Prevalence rates were compared by study year (or publication year if the study year was unavailable), country, subregion, diagnostic test, diagnostic criteria and values, and gender.

To address measurement heterogeneity, all summaries were stratified by diagnostic test, FBG, OGTT, casual (or random) blood glucose test (CBGT), or HbA1c and diagnostic criteria (Table 1) (WHO 1985/1999/2003–06/2011) [12,13,14,15,16].

### 2.5. Primary Effect Measure

The primary effect measure in this review was the prevalence of diabetes mellitus, expressed as the percentage of participants with diabetes (diagnosed and undiagnosed combined) in each included study population. Where available, we report the sex-specific prevalence (men, women, both) as well as the 95% confidence intervals provided in the original studies. We did not calculate pooled effect sizes because of substantial heterogeneity across populations, diagnostic tests, and time periods. Instead, we present prevalence estimates descriptively and stratify by country, region, sex, diagnostic test, and diagnostic criteria. Owing to substantial heterogeneity across populations, diagnostic methods, and study periods, results are only reported descriptively.

## 3. Results

### 3.1. Study Identification and Coverage

The search identified 2267 records (Table 2); 60 documents comprising 73 population-based studies met the inclusion criteria, representing 44,148 individuals from 16 countries in the Americas. Most participants were from North America, followed by Central and South America. No eligible population-based studies were identified from Caribbean nations or from several countries in South America (Guyana, Uruguay, Peru, and Venezuela) and Central America (Costa Rica and El Salvador).

### 3.2. Prevalence of Diabetes by Subregion and Population Composition

With some exceptions, a pronounced subregional gradient was observed, with substantially higher diabetes prevalence reported in North America, intermediate prevalence in Central America, and the lowest rates in South America. Extreme values were evident at both ends of the spectrum. The highest prevalence reported was 70% among Native American populations in Arizona in the mid-1990s, with similarly elevated estimates (approximately 40%) documented among Indigenous groups in Delaware, the Dakotas, and selected First Nations communities in Canada. In contrast, five studies conducted in Brazil, Chile, Colombia, Mexico, and Paraguay reported no cases of diabetes, representing the lower bound of observed prevalence. Among studies reporting cases, prevalence ranged from 1 to 70% in North America, 5 to 14% in Central America, and 1 to 29% in South America.

### 3.3. Temporal Distribution of Studies

Clear temporal patterns were evident. Studies reporting the highest prevalence values were concentrated in the 1990s, particularly in North America. In more recent decades, prevalence estimates showed fewer extremes but continued wide variability across populations. Only one eligible study was published after 2020, indicating a marked decline in recent research activity.

### 3.4. Indigenous Groups Represented

Across all studies, 111 distinct Indigenous groups were represented (Supplementary Table S1). Most studies focused on specific ethnic groups; however, broader population labels or unspecified ethnic groups—reported as Indigenous (seven studies) or Indigenous/mestizo (five studies)—were more common in national or multi-country surveys. The Pima Indians were the most frequently studied group, appearing in six studies conducted in the United States and Mexico. Most studies identified participants based on residence in defined reservations, tribes, or communities. These studies focused on narrowly defined Indigenous ethnic groups using geographic or other identification methods. In contrast, some studies applied broader criteria that included individuals with varying degrees of admixture.

### 3.5. Diagnostic Methods and Sampling

Most studies used venous blood samples analyzed in laboratory settings and fasting blood glucose (FBG) as the primary diagnostic test (Supplementary Table S2). HbA1c was used in 13% of studies. Nearly half of the studies employed more than one diagnostic test, most commonly combining FBG and the OGTT.

Methodological factors influenced reported prevalence estimates. Studies using random or cluster sampling yielded lower prevalence estimates than volunteer-based studies, a difference that was particularly pronounced in the United States. Diagnostic approaches varied widely across studies; however, heterogeneity in prevalence persisted even within strata defined by diagnostic method. Studies relying on volunteer-based invitations showed considerable heterogeneity in response rates (Supplementary Table S3).

### 3.6. Obesity and Diabetes Clustering

Most studies reported mean body mass index (BMI) or the prevalence of obesity (Supplementary Tables S4–S7). In general, higher prevalence of diabetes clustered in populations with higher BMI or obesity prevalence. A notable exception was observed in an Aymara population in Chile, where the prevalence of diabetes remained low despite a high mean BMI.

### 3.7. Heterogeneity and Subregional Contrast

Overall, the findings demonstrate substantial heterogeneity in the prevalence of diabetes among Indigenous populations of the Americas. This variability is characterized by strong subregional contrasts, extreme values in selected populations, temporal clustering of high prevalence estimates in earlier decades, and variation related to diagnostic tests and sampling methods.

## 4. Discussion

This review synthesizes 50 years of research (1975–2025) conducted across 16 countries in the Americas. Although earlier studies were identified, they were excluded because standardized diagnostic test and blood glucose thresholds—later formalized by the American Diabetes Data Group [78] in 1979 and the World Health Organization [13] in 1985—and modern population-based sampling methods were not consistently applied before the mid-1970s. Accordingly, 1975 was defined a priori as the lower time boundary to enhance methodological comparability.

Pioneering earlier studies conducted by Stein [79], Bennett [80], Henry [81], and Frohman [82] in the United States in the 1960s had reported unusually high prevalence of diabetes, ranging from 29% to 43%, among the Cherokee, Pima, Cocopah, and Seneca ethnic groups, respectively. These reports prompted speculation that similar patterns might emerge elsewhere. However, in contrast to the consistently high prevalence of diabetes reported among North American groups, this review suggests generally lower diabetes prevalence among Indigenous populations in Latin America [30,32,48,67,83], with the notable exception of the Xavante people in Brazil (28.8% in 2012) [68]. The extreme prevalence observed among the Pima has been attributed to interactions between genetic susceptibility and environmental factors, particularly nutrition and body mass index (BMI) [84]. Although risk factors are not fully addressed in this review, some Indigenous groups—such as the Aymara in Chile—exhibit low diabetes prevalence despite high obesity rates [41], further underscoring population-specific dynamics.

As shown in some of the reviewed studies [18,29,33,34], evidence dating back to 1971 by Bennett et al. [80] and later confirmed by Sugarman and Percy in 1989 [22] demonstrated that the prevalence of diabetes among North American Indigenous populations could be several-fold higher than in the general population. Subsequent studies, particularly south of the US border, have reported highly heterogeneous findings, with both higher [51,54,63,66] and lower [35,36,38,41,45,54] prevalence estimates.

Although full methodological details were not always available, our findings reveal marked variation across countries, ethnic groups, time periods, and the stringency of criteria used to define and ascertain Native American heritage. While most studies identified participants only based on residence in reservations, others relied on genetic markers [34,71], surnames [19,52], self-identification [5,6], or spoken language [2,7,8,9,10]. Whereas most studies did not report inclusion of mixed-ancestry individuals, others mentioned participants with partial Native ancestry [18,29,33,34] as well as participants that self-identified as Indigenous or *mestizos* [54,65,66,67,77]. This heterogeneity underscores the limitations of cross-study comparisons and highlights the inappropriateness of treating Indigenous populations as a single, homogeneous epidemiological category. Beyond the factors discussed above, the observed variation likely reflects the combined influence of genetic susceptibility, environmental and socioeconomic contexts, historical trajectories, and evolving diagnostic and sampling methodologies.

These findings align with personalized medicine and precision public health frameworks, as well as consensus recommendations from the American Diabetes Association and the European Association for the Study of Diabetes [85,86], which emphasize population-specific approaches rather than uniform strategies [85,86,87,88].

A major strength of this review is the integration of monographs and gray literature, providing a comprehensive synthesis of the prevalence of diabetes among Indigenous populations in the Americas. However, important limitations remain, including the lack of detailed analyses of BMI, socioeconomic conditions, dietary patterns, and other modifiable risk factors. Because of that, no meta-analysis or pooled estimates were calculated; instead, prevalence was described by ethnic group, country, sex, diagnostic test, and criteria, consistent with best practices for scoping reviews.

Finally, despite demographic recovery, cultural revitalization, and increased legal recognition of Indigenous peoples [8,9], research on diabetes epidemiology in these populations has declined in recent decades—possibly reflecting reduced funding following the peak of North American research activity in the 1970s–1990s—highlighting the need for renewed investment and sustained attention.

## 5. Conclusions

This review provides a comprehensive synthesis of the prevalence of diabetes among Indigenous populations in the Americas. The analysis spanned five decades and multiple methodological traditions. The evidence clearly demonstrates that diabetes prevalence varies widely across ethnic groups and regions, reinforcing the need to interpret estimates within their specific population, geographic, and historical contexts, rather than as representative of Indigenous peoples as a whole.

These findings support the adoption of population-specific surveillance and intervention strategies, consistent with personalized medicine and precision public health approaches. Future research would benefit from renewed investment in Indigenous diabetes epidemiology; greater methodological standardization; and improved reporting, definitions, and contextual determinants. Strengthening and sustaining this evidence base are essential to formulate culturally appropriate prevention, care, and policy responses that address persistent inequities while respecting the diversity of Indigenous populations across the Americas.

## Figures and Tables

**Table 1:** World Health Organization criteria for the diagnosis of diabetes mellitus, 1985, 2003–2006, and 2011.

Test	1985 WHO Criteria [12]	1999 WHO Criteria [13]	2003, 2006 WHO Criteria [14,15]	2011 WHO Criteria [16]
Fasting blood glucose (FBG)	≥140 mg/dL (7.8 mmol/L)	≥126 mg/dL (7.0 mmol/L)	≥126 mg/dL (7.0 mmol/L)	≥126 mg/dL (7.0 mmol/L)
Oral glucose tolerance test (OGTT)	≥200 mg/dL (11.1 mmol/L)	≥200 mg/dL (11.1 mmol/L)	≥200 mg/dL (11.1 mmol/L)	≥200 mg/dL (11.1 mmol/L)
Glycated hemoglobin (HbA1c)	-	-	-	≥6.5%

**Table 2:** Prevalence of diabetes mellitus in Indigenous populations of the Americas.

	Country	Site	Year	Age	N	Ethnicity	Test	Method	Value	Men	Women	Both
1	USA [18]	Arizona	1975	25+	1414	Pima	OGTT	Laboratory	≥200 mg/dL	32.6	37.2	34.9 **
2	Chile [19]	Community	1983	20+	510	Mapuche	OGTT	Laboratory	≥200 mg/dL	0.4	1.4	1.0
3	Canada [20]	Ontario	1986–1987	20–64	671	Cree, Ojibwa	FBG	Laboratory	≥126 mg/dL	10.0	11.0	
4	USA [21]	Tribe, Minnesota	1988	20+	346	Chippewa	FBG/CBG and OGTT ^1^	Laboratory	≥140 mg/dL ≥200 mg/dL	13.4	16.1	14.8
5	USA [22]	Arizona	1987	20–74	275	Navajo	FBG	Laboratory	≥140 mg/dL	9.1	10.5	9.9
6	USA [23]	Arizona	1988	20+	231	Navajo	FBG/OGTT	Laboratory	≥140 mg/dL ≥200 mg/dL	10.8	14.3	12.4
7	USA [24]	Rural reservation, Arizona	1989–1990	20–74	419	Navajo	FBG/OGTT	Laboratory	≥140 mg/dL ≥200 mg/dL	13.9 (9.2–18.5)	18.4 (14.0–22.8)	16.5 (13.2–19.7)
8	USA [25]	Multiple	1989–1991	45–74	3638	Akimel O’odham, Pee-Posh, Tohono O’odham, Ak-Chin Community, Apache, Ft. Sill Apache, Caddo, Comanche, Delaware, Kiowa, Wichita, Oglala Sioux, Cheyenne River Sioux, Spirit Lake Sioux	OGTT	Laboratory	≥200 mg/dL	42.1	52.7	
9	USA [26]	Tucson, Arizona	1990	25–65	230	Pascua Yaqui	FBG/OGTT ^2^	Laboratory	≥140 mg/dL ≥200 mg/dL	35.4	38.9	
10	USA [23]	Rough Rock community	1992	20+	827	Navajo	FBG/OGTT	Laboratory	≥140 mg/dL ≥200 mg/dL	10.8 (4.4, 17.2)	14.3 (8.5, 20.1)	12.4 (8.1, 16.7)
11	USA [27]	Southwestern US	1991-92	20+	575	Navajo	FBG/OGTT	Laboratory	≥140 mg/dL ≥200 mg/dL	19.4	24.6	22.9
12	USA [28]	Minnesota	1992–1994	25+	981	Chippewa and Menominee	FBG/OGTT	Laboratory	≥140 mg/dL ≥200 mg/dL	27.00	29.00	28.00
13	Mexico [29]	Community, Sonora	1994	20+	224	Pima	FBG/OGTT	Laboratory	≥140 mg/dL ≥200 mg/dL	5.6	8.5	6.9
14	USA [29]	Arizona	1995	20+	888	Pima	FBG/OGTT	Laboratory	≥140 mg/dL ≥200 mg/dL	34.2	40.8	38.0
15	Canada [30]	River Desert, Quebec	1995	30–64	131	Algonquins	FBG/OGTT	Laboratory	≥140 mg/dL ≥200 mg/dL	16.3 (7.9–24.7)	16.3 (9.0–23.6)	
16	Canada [30]	Lac Simon. Quebec	1995	30–64	168	Algonquins	FBG/OGTT	Laboratory	≥140 mg/dL ≥200 mg/dL	23.9 (12.9–34.9)	48.6 (38.4–58.8)	
17	USA [25]	Multiple	1993–1995	45–74	3638	Akimel O’odham, Pee-Posh, Tohono O’odham, Ak-Chin Community, Apache, Ft. Sill Apache, Caddo, Comanche, Delaware, Kiowa, Wichita, Oglala Sioux, Cheyenne River Sioux, Spirit Lake Sioux	OGTT	Laboratory	≥200 mg/dL	48.2	61.3	
18	Canada [31]	Sandy Lake, Ontario	1993–1995	20+ **	728	Ojibwa-Cree	FBG/OGTT	Laboratory	≥140 mg/dL ≥200 mg/dL	28.0	24.2	26.1
19	USA [32]	Arizona	1995	20–74	900	Pima, Maricopa, Papago	FBG/CBG/OGTT ^3^	Laboratory	≥140 mg/dL ≥200 mg/dL	65 (60.3–69.1)	72 (69.3–75.3)	70 (67.2–72.2)
20	USA [32]	Delaware	1995	20–74	400	Apache, Caddo, Comanche	FBG/CBG/OGTT ^3^	Laboratory	≥140 mg/dL ≥200 mg/dL	38 (33.9–41.8)	42 (38.6–45.4)	40 (37.7–42.8)
21	USA [32]	N and S Dakota	1995	20–74	200	Ogala, Sioux, Cheyenne, River Sioux, Devils Lake Sioux	FBG/CBG/OGTT ^3^	Laboratory	≥140 mg/dL ≥200 mg/dL	33 (29.0–36.8)	46 (42.1–49.1)	40 (37.5–42.8)
22	Mexico [33,34]	Maycoba, Sonora	1995	20+	226	Pima	FBG/OGTT	Laboratory	≥126 mg/dL ≥200 mg/dL	5.6	8.5	7.1
23	Mexico [35,36]	Durango	1996	30–64	193	Tepehuanos, Huicholes, Mexicaneros	FBG/OGTT	Glucometer	≥140 mg/dL ≥200 mg/dL	0.0	0.0	0.0
24	Mexico [37]	Community	1996–1997	15–77	91	Otomíes	FBG	Laboratory	≥126 mg/dL	-	-	4.4 (0.1–8.7)
25	Colombia [38]	Rural	1996 *	18+	147	Arhuaco, Arzario, Kogui, Wayuu	OGTT	Glucometer	>10 mmol/L	0.0	0.0	0.0
26	Bolivia [39]	Urban El Alto	1998	25+	776	Aymara	OGTT	Glucometer	≥200 mg/dL	5.3 (2.2–8.3)	4.3 (2.7–6.7)	4.8 (3.4–6.6)
27	Bolivia [39]	Urban Cochabamba	1998	25+	266	Quechua	OGTT	Glucometer	≥200 mg/dL	7.9 (4.2–14.3)	8.9 (5.2–14.8)	8.4 (5.6–12.4)
28	Chile [40]	Rural Community	1998	20+	319	Mapuche	OGTT	Laboratory	≥200 mg/dL	3.2 (0.7–9.0)	4.5 (2.2–8.1)	4.1 (2.2–6.9)
29	Chile [41]	Rural Community	1998	20+	196	Aymara	OGTT	Laboratory	≥200 mg/dL	1.3 (0.0–7.0)	1.7 (0.2–6.0)	1.5 (0.3–4.5)
30	Paraguay [42]	Community, Chaco	1998	18–70	225	Ayoreos	FBG or OGTT	Laboratory	≥126 mg/dL ≥200 mg/dL	0.0	0.0	0.0
31	Guatemala [43]	Rural, Urban Sololá	1998	30+	400	Quiche, Cakchiquel, Zutuhil	OGTT	Laboratory	≥200 mg/dL	-	-	6.8
32	Brazil [44]	Parque Indígena do Xingu (Mato Grosso)	1999	20+	86	Suyá	FBG	Glucometer	≥126 mg/dL	0.0	0.0	0.0
33	Mexico [45]	Community, Merida	2000	18–81	263	Maya	FBG	Laboratory	≥126 mg/dL	-	-	10.6
34	Brazil [46]	Pará	2000	20+	122	Parkateje	FBG or OGTT	Glucometer	≥126 mg/dL ≥200 mg/dL	-	-	1.1
35	Mexico [47]	National	2000	20+	3645	Multiple indigenous groups	FBG/A1c	Laboratory	≥126 mg/dL or 6.5%			4.1 (3.1, 5.2)
36	Canada [48,49]	Sandy Bay Ojibway First Nation, Manitoba	2002–2003	18+	482	Ojibway	FBG	Laboratory	≥126 mg/dL	27.0 (21.2–32.7)	31.0 (25.2–36.7)	29.0 (25.0–33.1)
37	Brazil [50]	Upper Xingu	2002–2003	20+	251	Kalapalo, Kuikuro, Matipu, Nahukwá	FBG	Laboratory	≥126 mg/dL	0.0	0.0	0.0
38	Brazil [51]	Community, Espírito Santo	2003–2004	25–64	620	Guarani, Tupinikin	FBG	Laboratory	≥126 mg/dL	2.4	2.7	-
39	Chile [52]	Santiago and Arica	2004 *	18+	160	Aymara	OGTT	Laboratory	≥200 mg/dL	2.4 (0.1–12.6)	8.5 (4.1–15.0)	
40	Chile [52]	Santiago and Arica	2004 **	18+	147	Mapuche	OGTT	Laboratory	≥200 mg/dL	14.3 (5.4–28.5)	5.7 (201–12.0)	
41	Canada [53]	Mistissini, Quebec	2005	18+	172	Cree	FBG	Laboratory	≥126 mg/dL			20.0
42	Belice [54]	National	2003–2006	20+	1192	Indigenous/ Mestizo	FBG or OGTT	Laboratory	≥126 mg/dL ≥200 mg/dL	8.6 (6.0–12.1)	13.6 (11.1–16.4)	11.0 (9.0–13.5)
43	Guatemala [54]	Urban, Guatemala City	2003–2006	20+	1395	Indigenous/ Mestizo	FBG or OGTT	Laboratory	≥126 mg/dL ≥200 mg/dL	7.8 (5.1–11.8)	6.8 (4.8–9.4)	7.3 (5.4–9.7)
44	Honduras [54]	Urban, Tegucigalpa	2003–2006	20+	1592	Indigenous/ Mestizo	FBG or OGTT	Laboratory	≥126 mg/dL ≥200 mg/dL	5.0 (2.7–9.1)	5.3 (3.8–7.2)	5.1 (3.2–8.0)
45	Nicaragua [54]	Urban, Managua	2003–2006	20+	1530	Indigenous/ Mestizo	FBG or OGTT	Laboratory	≥126 mg/dL ≥200 mg/dL	9.5 (7.2–12.5)	10.8 (8.1–14.2)	10.2 (8.2–12.6)
46	Mexico [47]	National	2006	20+	513	Multiple indigenous groups	FBG HbA1c	Laboratory	≥126 mg/dL or 6.5%			9.4 (6.8, 12.9)
47	Mexico [55]	Community, Sonora	2006	20–65	120	Yaquis	FBG OGTT	Laboratory	≥126 mg/dL ≥200 mg/dL	20.5	17.6	18.3
48	Mexico [55]	Community, Sonora	2006–2007	20–65	158	Tepehuanos	FBG OGTT	Laboratory	≥126 mg/dL ≥200 mg/dL	-	-	0.8
49	Brazil [56,57]	Community, Jaguapiru, Mato Grosso do Sul	2007–2008	18–69	606	Guarani, Kaiowa, Terena	FBG/OGTT ^4^	Glucometer	≥126 mg/dL ≥200 mg/dL	1.5	6.8	4.5
50	Mexico [58]	Community, Oaxaca	2010 *	35+	394	Zapotec	FBG/OGTT	Not mentioned	≥126 mg/dL ≥200 mg/dL	6.2	13.3	8.7
51	Mexico [58]	Community, Oaxaca	2010 *	35+	730	Mixe	FBG/OGTT	Not mentioned	≥126 mg/dL ≥200 mg/dL	5.7	7.1	6.9
52	Colombia [59]	Communities, Caldas	2010	20–69	151	Embera-Chamí	FBG	Laboratory	≥126 mg/dL			7.9
53	Mexico [33,34]	Maycoba, Sonora	2010	20+	359	Pima	FBG/OGTT	Laboratory	≥126 mg/dL ≥200 mg/dL	6.0	11.8	9.0
54	Brazil [60]	Xingu Indigenous Park	2010–2011	20+	181	Khisêdjê	OGTT	Laboratory	≥200 mg/dL	2.0	6.8	3.8
55	Chile [61]	Rural	2011	15+	264	Pehuenche	FBG ^5^	Laboratory	≥126 mg/dL ≥200 mg/dL	-	-	0.8
56	Panama [62]	Provinces of Panama and Colon	2011	18+	203	Native American	FBG/HbA1c	Laboratory	≥126 mg/dL 6.5%			5.4 (3.2–7.6)
57	Mexico [63]	Urban/rural, Chiapas	2010–2012	20+	880	Indigenous	FBG/OGTT	Laboratory	≥126 mg/dL ≥200 mg/dL	3.5 (1.6–5.5)	5.8 (3.8–7.8)	4.7 (3.3–6.1)
58	Canada [48,64]	Sandy Bay Ojibway First Nation, Manitoba	2011–2012	18+	596	Ojibway	FBG	Laboratory	≥126 mg/dL	24.8 (20.0–29.6)	27.1 (21.9–32.3)	25.9 (22.4–29.4)
59	Mexico [47]	National	2012	20+	1122	Multiple indigenous groups	FBG HbA1c	Laboratory	≥126 mg/dL or 6.5%			12.7 (9.8, 16.3)
60	Guatemala [65]	Community, Atitlán	2012–2013	20–65	308	Tzu’tujil, Kaqchikel, mestizo	FBG	Glucometer	≥126 mg/dL	3.0 (1.1–4.8)	1.3 (0.0–3.2)	4.6 (1.6, 7.7)
61	Suriname [66]	National	2013	15–65	279	Amerindian	FBG	Laboratory	≥126 mg/dL	15.8	8.7	13.0
62	Mexico [67]	Community Baja California	2013–2014	18+	275	Indigenous/mestizo	HbA1c	Micro method	≥6.5%	18.6	22.9	21.8
63	Brazil [68]	Community, Mato Grosso	2010–2012	20+	948	Xavante	OGTT	Glucometer	≥200 mg/dL	18.4 (14.9–22.2)	40.6 (36.2–45.1)	28.8 (25.3–31.1)
64	Argentina [69]	Communities, Chaco	2014	18+	156	Wichi	CBGT	Glucometer	≥200 mg/dL	1.7	0.0	0.6
65	Guatemala [70]	Community, Atitlán	2015	18+	394	Tzu’tujil, Kaqchikel	HbA1c	Glucometer	≥6.5%	12.2 (6.3–8.1)	14.6 (10.3–18.8)	13.8 (10.4–17.2)
66	Colombia [71]	Cristiania (Jardín) Antioquia	2015 *	14+	145	Embera-Chamí	FBG	Not mentioned	≥126 mg/dL	0.0	0.9	0.7
67	Brazil [72]	Communities	2018	18+	459	Munduruku	FBG	Glucometer	≥126 mg/dL			12.2
68	Brazil [73]	Community, Amazonia	2016	18+	455	Mura	FBG	Glucometer	≥126 mg/dL			3.0 (1.8–5.1)
69	Mexico [47]	National	2018	20+	1177	Multiple indigenous groups	FBG/HbA1c ^6^	Laboratory	≥126 mg/dL or 6.5%			18.7 (15.0, 23.2)
70	Guatemala [74]	Rural	2018–2019	18+	640	Maya	A1c	Micro method	≥6.5%	12.2 (7.4–16.9)	12.9 (9.4–16.4)	12.2 (7.4–16.9)
71	Panama [75]	Community	2019 *	18+	211	Kuna Indians	A1c	Micro method	≥6.5%	14.0	12.9	13.0
72	Brazil [76]	Community, Pará	2019	18+	363	Xikrin (Mebengôkre)	FBG	Laboratory	≥126 mg/dL	4.4	4.9	3.8
73	Ecuador [77]	Municipality	2022	18+	111	Chachi	FBG/OGTT ^7^	Laboratory	≥126 mg/dL or ≥200 mg/dL			0.9

(*) Publication year; (**) Truncated rates calculated from original data. FBG (fasting blood glucose); OGTT (oral glucose tolerance test); CBGT (casual blood glucose test); HbA1c (glycated hemoglobin). CBG: Casual Blood Glucose; FBG/HbA1c: Fasting Blood Glucose/ Glicated Hemoglobine; HbA1: Glicated Hemoglobine; HbA1c: Glicated Hemoglobine; HBA1: Glicated Hemoglobine. ^1^ OGTT if FBG ≥ 115 mg/dL or CBG ≥ 140 mg/dL and FBG ≥ 126 mg/dL; ^2^ OGTT with 100 g of glucose; ^3^ FBG ≥ 140 mg/dL or OGTT ≥ 200; ^4^ (FBG ≥ 126 mg/dL on two occasions, OGTT if FBG ≥ 100 mg/dL and <126 mg/dL; ^5^ Two FBG ≥ 126 mg/dL, or one FBG ≥ 200 mg/dL; ^6^ FBG ≥126 mg/dL or A1c ≥ 6.5%; ^7^ FBG ≥ 144 mg/dL, OGTT if FBG < 144 mg/dL.

## Data Availability

The original contributions presented in this study are included in the article and Supplementary Materials. Further inquiries can be directed to the corresponding author.

## Supplementary Materials

The following supporting information can be downloaded at: https://www.mdpi.com/article/10.3390/jpm16020116/s1, Supplementary Table S1. Ethnic groups represented in studies of diabetes among Indigenous in the Americas by country; Supplementary Table S2. Diagnosis test used for the studies of diabetes in Indigenous populations of the Americas by country; Supplementary Table S3. Response rate (%) to open invitations by studies of diabetes in Indigenous populations in the Americas; Supplementary Table S4. Body Mass Index and the prevalence of diabetes among Indigenous populations of the Americas among males; Supplementary Table S5. Body Mass Index and the prevalence of diabetes among Indigenous populations of the Americas among females; Supplementary Table S6. Body Mass Index and the prevalence of diabetes among Indigenous populations of the Americas among males and females; Supplementary Table S7. Obesity (%) and the prevalence of diabetes (%) by study and gender in Indigenous populations of the Americas; Supplementary Table S8: PRISMA checklist.
