# Supplementary material for: Population Heterogeneity of Diabetes in Indigenous Peoples of the Americas: A Systematic Scoping Review of the Existing Literature
Source: J Pers Med. 2026 Feb 14;16(2):116. doi: 10.3390/jpm16020116 (PMC12941647; doi:10.3390/jpm16020116)
Supplement: Supplementary file 1 [file jpm-16-00116-s001.zip › jpm-4058772-supplementary materials (S1-S7).pdf]

Table S1. Diagnosis test used for the studies of diabetes in indigenous population of the Americas by country.

	OGTT	FBG	FBG+OGTT	CBG	A1C	FBG+A1C	FBG+CBG	Total
Argentina	0	0	0	1	0	0	0	1
Belice	0	0	1	0	0	0	0	1
Bolivia	2	0	0	0	0	0	0	2
Brazil	1	6	3	0	0	0	0	10
Canada	0	4	3	0	0	0	0	7
Chile	5	0	0	0	0	0	1	6
Colombia	1	2	0	0	0	0	0	3
Ecuador	0	0	1	0	0	0	0	1
Guatemala	0	1	2	0	2	0	0	5
Honduras	0	0	1	0	0	0	0	1
Mexico	0	2	9	0	1	4	0	16
Nicaragua	0	0	1	0	0	0	0	1
Panama	0	0	0	0	1	1	0	2
Paraguay	0	0	1	0	0	0	0	1
Surinam	0	1	0	0	0	0	0	1
USA	3	0	8	0	0	0	4	15
Total	12	16	30	1	4	5	5	73
%	16.4	21.9	41.1	1.4	5.5	6.8	6.8	100.0

Table S2. Ethnic groups represented in studies of diabetes among indigenous in the Americas by country.

COUNTRY	ETHNIA	STUDIES
**Argentina**	**Wichi**	**1**
Bolivia	Aymara	1
Bolivia	Quechua	1
**Bolivia**	**Subotal**	**2**
Brazil	Suyá	1
Brazil	Parkateje	1
Brazil	Guarani	1
Brazil	Tupinikin	1
Brazil	Guarani	1
Brazil	Kaiowa	1
Brazil	Terena	1
Brazil	Xavante	1
Brazil	Munduruku	1
Brazil	Mura	1
Brazil	Xikrin (Mebengôkre)	1
**Brazil**	**Subotal**	**11**
Canada	Algonquins	1
Canada	Algonquins	1
Canada	Ojibwa-Cree	1
Canada	Ojibway	1
Canada	Cree	1
Canada	Ojibway	1
Canada	Total	6
Chile	Mapuche	1
Chile	Aymara	1
Chile	Aymara	1
Chile	Mapuche	1
Chile	Pehuenche	1
Chile	Mapuche	1
**Chile**	**Total**	**6**
Colombia	Arhuaco	1
Colombia	Arzario	1
Colombia	Kogui	1
Colombia	Embera-Chamí	1
Colombia	Embera-Chamí	1
Colombia	Wayuu	1
**Colombia**	**Total**	**6**
Ecuador	Chachi	1
Guatemala	Quiche	1
Guatemala	Cakchiquel	1
Guatemala	Zutuhil	1
Guatemala	Tzu'tujil	1
Guatemala	Kaqchikel	1
Guatemala	Tzu'tujil	1
Guatemala	Kaqchikel	1
Guatemala	Maya	1

**Guatemala**	**Subotal**	**9**
Mexico	Pima	1
Mexico	Pima	1
Mexico	Huicholes	1
Mexico	Mexicaneros	1
Mexico	Otomíes	1
Mexico	Maya	1
Mexico	Yaquis	1
Mexico	Tepehuanos	1
Mexico	Zapotec	1
Mexico	Mixe	1
Mexico	Pima	1
Mexico	Maya	1
Mexico	Triqui	1
Mexico	Zapotec	1
Mexico	Tepehuanos	1
Mexico	Mixtec	1
Mexico	Total	16
Panama	Emberá, Guna	1
Panama	Kuna Indians	1
**Panama**	**Subtotal**	**2**
**Paraguay**	**Ayoreos**	**1**
USA	Akimel O'odham	1
USA	Pee-Posh	1
USA	Tohono O'odham	1
USA	Ak-Chin Community	1
USA	Apache	1
USA	Ft. Sill Apache	1
USA	Caddo	1
USA	Comanche	1
USA	Delaware	1
USA	Kiowa	1
USA	Wichita	1
USA	Oglala Sioux	1
USA	Cheyenne River Sioux	1
USA	Spirit Lake Sioux	1
USA	Akimel O'odham	1
USA	Pee-Posh	1
USA	Tohono O'odham	1
USA	Ak-Chin Community	1
USA	Apache	1
USA	Ft. Sill Apache	1
USA	Caddo	1
USA	Comanche	1
USA	Delaware	1
USA	Kiowa	1
USA	Wichita	1
USA	Oglala Sioux	1
USA	Cheyenne River Sioux	1

USA	Spirit Lake Sioux	1
USA	Pima	1
USA	Navajo	1
USA	Pima	1
USA	Pima	1
USA	Maricopa	1
USA	Apache,	1
USA	Caddo,	1
USA	Comanche	1
USA	Ogala	1
USA	Sioux	1
USA	Cheyenne	1
USA	River Sioux	1
USA	Devils Lake Sioux	1
USA	Chippewa	1
USA	Navajo	1
USA	Navajo	1
USA	Papago	1
**USA**	**Total**	**48**
All	Overall	111

Table S3. Body Mass Index and the prevalence of diabetes among indigenous populations of the Americas among males.

Study (country, ethnic group, author, year)	DM prevalence	Mean BMI
USA, Pima, Maricopa, Papago, Lee 1995 [32]	65.0	31.10
USA, O'odham–Pee Posh peoples (Arizona); Southwestern–Plains Tribes (Oklahoma); and Sioux Nation (Dakotas), Welty 1995 [25]	48.2	29.80
USA, O'odham–Pee Posh peoples (Arizona); Southwestern–Plains Tribes (Oklahoma); and Sioux Nation (Dakotas), Welty 1991 [25]	42.1	29.90
USA, Apache, Caddo, Comanche, Lee 1995 [32]	38.0	30.20
USA, Ogala, Sioux, Cheyenne, River Sioux, Devils Lake Sioux, Lee 1995 [32]	33.0	28.50
USA, Chippewa, Menominee; Casper 1996 [28]	27.0	30.40
Canada, Ojibway, Bruce 2003 [49]	27.0	29.65
Mexico, Yaquis, Rodriguez 2006 [55]	20.5	29.40
Mexico, Indigenous/mestizo, Pacheco 2014 [67]	18.6	27.10
Brazil, Xavante, Dal Fabbro 2014 [68]	18.4	30.70
Chile, Mapuche, Carrasco 2004 [52]	14.3	29.80
USA, Navajo, Sugarman 1987 [22]	13.9	27.70
USA, Navajo, Hall 1992 [23]	10.8	27.70
Nicaragua, Indigenous/mestizo, Barcelo 2006 [54]	9.5	26.60
USA, Navajo; Sugarman 1989 [24]	9.1	27.70
Belice, Indigenous/mestizo, Barcelo 2006 [54]	8.6	27.00
Guatemala, Indigenous/mestizo, Barcelo 2006 [54]	7.8	25.70
Mexico, Pima, Esparza 1995 [33]	5.6	23.80
Mexico, Pima, Schulz 1996 [29]	5.6	23.80
Honduras, Indigenous/mestizo, Barcelo 2006 [54]	5.0	25.90
Brazil, Xikrin (Mebengôkre), Barbosa 2019 [76]	4.4	27.70
Guatemala, Tzu'tujil, Kaqchikel & mestizo, Chen 2013 [65]	3.0	12.40
Brazil, Guarani & Tupinikin, Alvim 2014 [51]	2.4	24.60
Chile, Aymara, Carrasco 2004 [52]	2.4	28.20
Argentina, Wichi, Bianchi 2014 [69]	1.7	28.40
Chile, Aymara, Santos 1998 [41]	1.3	24.90
Mexico, Tepehuanos, Rodriguez 2007 [55]	0.0	22.10
Brazil, Suyá, De Salvo 1999 [44]	0.0	23.50

Table S4. Body Mass Index and the prevalence of diabetes among indigenous populations of the Americas among females.

Study (country, ethnic group, author, year)	DM prevalence	Mean BMI
USA, Pima, Maricopa, Papago, Lee 1995 [32]	72.0	33.10
USA, O'odham–Pee Posh peoples (Arizona); Southwestern–Plains Tribes (Oklahoma); and Sioux Nation (Dakotas), Welty 1995 [25]	61.3	31.70
USA, O'odham–Pee Posh peoples (Arizona); Southwestern–Plains Tribes (Oklahoma); and Sioux Nation (Dakotas), Welty 1991 [25]	52.7	31.80
USA, Ogala, Sioux, Cheyenne, River Sioux, Devils Lake Sioux, Lee 1995 [32]	46.0	30.10
USA, Apache, Caddo, Comanche, Lee 1995 [32]	42.0	31.30
Brazil, Xavante, Dal Fabbro 2014 [68]	40.6	29.70
Canada, Ojibway, Bruce 2003 [49]	31.0	33.32
USA, Chippewa, Menominee; Casper 1996 [28]	29.0	30.80
Mexico, Indigenous/mestizo, Pacheco 2014 [67]	22.9	29.20
USA, Navajo, Sugarman 1987 [22]	18.4	28.20
Mexico, Yaquis, Rodriguez 2006 [55]	17.6	30.00
USA, Navajo, Hall 1992 [23]	14.3	26.00
Belice, Indigenous/mestizo, Barcelo 2006 [54]	13.6	29.40
Nicaragua, Indigenous/mestizo, Barcelo 2006 [54]	10.8	29.00
USA, Navajo; Sugarman 1989 [24]	10.5	28.20
Mexico, Pima, Esparza 1995 [33]	8.5	26.00
Mexico, Pima, Schulz 1996 [29]	8.5	26.30
Chile, Aymara, Carrasco 2004 [52]	8.5	30.10
Guatemala, Indigenous/mestizo, Barcelo 2006 [54]	6.8	27.30
Chile, Mapuche, Carrasco 2004 [52]	5.7	31.80
Honduras, Indigenous/mestizo, Barcelo 2006 [54]	5.3	27.40
Brazil, Xikrin (Mebengôkre), Barbosa 2019 [76]	4.9	29.60
Brazil, Guarani & Tupinikin, Alvim 2014 [51]	2.7	26.20
Chile, Aymara, Santos 1998 [41]	1.7	26.70
Mexico, Tepehuanos, Rodriguez 2007 [55]	1.3	24.20
Guatemala, Tzu'tujil, Kaqchikel & mestizo, Chen 2013 [65]	1.3	37.30
Brazil, Suyá, De Salvo 1999 [44]	0.0	25.70
Argentina, Wichi, Bianchi 2014 [69]	0.0	27.20

Table S5. Body Mass Index and the prevalence of diabetes among indigenous populations of the Americas among males and females.

Study (country, ethnic group, author, year)	DM prevalence	Mean BMI
Brazil, Xavante, Dal Fabbro 2014 [68]	28.8	30.30
USA, Chippewa, Menominee; Casper 1996 [28]	28.0	30.60
Mexico, Indigenous/mestizo, Pacheco 2014 [67]	21.8	28.70
Canada, Cree, Chateau 2005 [53]	20.0	32.60
USA, Navajo; Hall 1992 [23]	14.3	26.00
Brazil, Munduruku, Gomes 2018 [72]	12.2	25.80
Belice, Indigenous/mestizo, Barcelo 2006 [54]	11.0	28.20
Mexico, Maya, Loria 2000 [45]	10.6	28.80
Nicaragua, Indigenous/mestizo, Barcelo 2006 [54]	10.2	26.80
Brazil, Mura, De Souza 2018 [73]	8.1	26.65
Colombia, Embera Chamí, Cardona 2010 [59]	7.9	26.80
Guatemala, Indigenous/mestizo, Barcelo 2006 [54]	7.3	28.40
Honduras, Indigenous/mestizo, Barcelo 2006 [54]	5.1	26.80
Mexico, Indigenous, Jimenez 2012 [63]	4.7	25.50
Guatemala, Tzu'tujil, Kaqchikel & mestizo, Chen 2013 [65]	4.6	25.20
Brazil, Xikrin (Mebengôkre), Barbosa 2019 [76]	3.8	28.80
Argentina, Wichi, Bianchi 2014 [69]	0.6	27.90
Colombia, Arhuaco, Arzario, Kogui & Wayuu, Briceno 1996 [38]	0.0	22.90
Mexico, Tepehuanos, Huicholes, Mexicaneros, Guerrero 1996 [36]	0.0	24.40

Table S6. Obesity (%) and the prevalence of diabetes (%) by study and gender in indigenous populations of the Americas.

Study (country, ethnic group, author, year)	Diabetes	Obesity
Males		
USA, Pasqu Yaquis; Campos-Outcal 1995 [26]	35.4	40.00
Canada, Ojibway, Riediger 2012 [48]	24.8	35.60
Panama, Kuna Indians, Campbell 2019 [75]	14.0	30.20
Guatemala, Maya, Steinbrook 2019 [74]	12.2	17.00
USA, Cree, Ojibwa; Young 1990 [20]	10.0	5.55
Colombia, Embera-Chamí, Cataño Bedoya 2015 [71]	0.0	3.00
Females		
USA, Pasqu Yaquis; Campos-Outcal 1995 [26]	38.9	69.00
Canada, Ojibway, Riediger 2012 [48]	27.1	60.20
Guatemala, Maya, Steinbrook 2019 [74]	12.9	30.10
Panama, Kuna Indians, Campbell 2019 [75]	12.9	40.80
USA, Cree, Ojibwa; Young 1990 [20]	11.0	5.53
Colombia, Embera-Chamí, Cataño Bedoya 2015 [71]	0.9	10.60
Both genders		
Mexico, Indigenous, Castro 2018 [47]	18.7	52.10
Guatemala, Tzu'tujil, Kaqchikel, Bream 2015 [70]	13.8	26.70
Mexico, Indigenous, Castro 2012 [47]	12.7	37.80
Mexico, Indigenous, Castro 2006 [47]	9.4	40.20
Mexico, Indigenous, Castro 2000 [47]	4.1	12.30
Chile, Pehuenche, Navarrete 2011 [61]	0.8	28.10
Colombia, Embera-Chamí, Cataño Bedoya 2015 [71]	0.7	8.00
Chile, Mestizo, Navarrete 2011 [61]	0.0	28.40

Table S7. Response rate (%) to open invitations by studies of diabetes in indigenous populations in the Americas.

Study	Response Rate (%)
Brazil;Espirito Santo;Guarani & Tupinikin;Alvim 2014 [51]	71
Brazil;Para;Parkateje;Tavares 2000 [46]	90
Brazil;Xingu Indigenous Park;Khisêdjê;Santos 2011 [60]	95
Canada;Manitoba;Ojibway;Bruce 2003 [49]	36
Canada;Manitoba;Ojibway;Riediger 2012 [48]	44
Canada;Ontario;Algonquian-speaking Ojibwa-Cree;Harris 1995 [31]	70
Colombia;Rural;Arhuaco, Arzario, Kogui & Wayuu;Briceno 1996 [38]	48
Mexico;Chiapas;Indigenous;Jimenez 2012 [63]	74
Mexico;Community;Otomíes;Alvarado 1997 [37]	80
Mexico;Community;Pima;Schulz 1996 [29]	77
Mexico;Maycoba, Sonora;Pima;Esparza 1995 [33]	78
Mexico;Maycoba, Sonora;Pima;Esparza 2010 [33]	86
Mexico;Merida;Maya;Loria 2000 [45]	25
Paraguay;Chaco;Ayoreos;Benitez 1998 [42]	91
USA;Arizona;Navajo;Sugarman 1989 [24]	65
USA;Arizona;Pima, Maricopa, Papago;Lee 1995 [32]	71
USA;Arizona;Pima;Knowler 1975 [18]	88
USA;Delaware;Apache, Caddo, Comanche;Lee 1995 [32]	53
USA;N & S Dakota;Ogala, Sioux, Cheyenne, River Sioux, Devils Lake Sioux;Lee 1995 [32]	62
